# OA02 Blood, Sweat and Steroids

**DOI:** 10.1093/rap/rkae117.002

**Published:** 2024-11-05

**Authors:** Sarah Stewart, David McCormick

**Affiliations:** Ulster Hospital, Belfast, United Kingdom; Ulster Hospital, Belfast, United Kingdom

## Abstract

**Introduction:**

This 79 year old gentleman was referred to the Rheumatology department with arthralgia, myalgia, erythema nodosum, an ESR elevated at 106 mm/h and IgA 6.45 g/L. His GP commenced him on 30 mg Prednisolone once a day for one week. His symptoms and signs improved significantly with treatment, prior to his first Rheumatology review.

**Case description:**

He initially presented to his GP with pain and a skin rash in his groin which was thought to be fungal and treated as such. He then developed erythematous tender lumps on his right proximal forearm and left hand which were in keeping with erythema nodosum. He had a background history of ischaemic heart disease, hypertension, hyperlipidaemia, squamous cell carcinoma and an elevated BMI.

Once the initial course of prednisolone stopped he experienced recurrence of the skin rash, in addition to night sweats, dizziness and weight loss. Inflammatory markers rose.

CT scan of chest, abdomen and pelvis, done to exclude malignancy, was normal. PET CT while off steroid showed uptake in the descending aorta, not in keeping with large vessel vasculitis, and generalised bone marrow uptake consistent with an inflammatory process. Prednisolone was re-commenced. Bloods including HIV, Hepatitis, B12, CMV, EBV, Syphilis, ANA, ANCA and Cryoglobulins were normal. Skin biopsy showed non-specific inflammatory change. Bone marrow biopsy showed myeloid hyperplasia and haemophagocytosis.

He required two further inpatient admissions due to shortness of breath, anaemia and skin rash, which coincided with steroid reduction. He responded to an increased dose of steroid and antibiotics.

Genetic testing then identified a gene for UBA1, a pathogenic variant causing VEXAS syndrome. Clinical features were in keeping – skin lesions, sweats, elevated inflammatory markers, macrocytic anaemia and steroid responsivity.

He was commenced on Tocilizumab and responded well. Bloods normalised and prednisolone was weaned. Neutropenia resulted in stopping Tocilizumab and later Anakinra and Sarilumab. Half dose once monthly Tocilizumab and low dose steroid are now successfully treating his disease.

**Discussion:**

This gentleman had symptoms and signs in keeping with an auto-inflammatory condition. However, it took a number of specialties to investigate him due to blood results and imaging not naturally leading to a diagnosis. Initial differentials included sarcoid and polyarteritis nodosa. The chest and joints were not involved, serum ACE, calcium and CT Angio were normal so these were excluded. PET CT was done to ensure this was not an atypical large vessel vasculitis and the abnormal bone marrow uptake led us to discuss with haematology. At that time he had a pancytopenia with poor reticulocyte response and so haematology carried out a bone marrow biopsy. The bone marrow biopsy result showed a myeloid shift felt to be reactive and there was a degree of haemophagocytosis but he did not meet diagnostic criteria for haemophagocytic lymphohistiocytosis. Still’s disease was queried but he was not pyrexic, there was no arthritis, no hepatomegaly or splenomegaly and there was neutropenia rather than a neutrophilia. Skin biopsy, while not diagnostic, did query a vasculitic process. Therefore it was decided, despite persisting diagnostic uncertainty that he should be treated with Tocilizumb, which did allow steroid reduction. Eventually genetic testing was sent looking for periodic fevers and that is when the UBA1 gene was identified and the diagnosis of VEXAS syndrome made. Subcutaneous Tocilizumab did cause leucopenia and he suffered from recurrent infections. He had also been trialled on Anakinra and Sarilumab but both of these caused neutropenia. We have been able to switch to intravenous Tocilizumab in order to titrate the dose, and he is now on a small dose of 2 mg/kg 4 weekly which has allowed inflammation to settle without causing a drop in white cell count.

**Key learning points:**

• There is little guidance on treatment of VEXAS syndrome. This gentleman has been difficult to treat due to drug side effects, and so this case raises the issue of few treatment options in VEXAS. It seems little is known about the disease and so we quickly come to a treatment conundrum when side effects prohibit the use of standard therapies. More research is required in to this condition and its management.

• This case also raises the importance of working closely with other specialties. Where there is multi-system involvement, multi-disciplinary working is invaluable. It brings other perspectives and breadth of knowledge to the case, increasing the likelihood of making the correct diagnosis.

• This case also reminds us of the value of genetic testing. Where it seems there is an inflammatory syndrome but results don’t seem to add up, we should put our patients forward for testing.

• At the BSR case based conference I would like to hear other cases of treating VEXAS and what difficulties and wisdom my colleagues have to share. I would also like to hear if there are other treatment strategies others are aware of.

